# Morphometry of the Brachiocephalic Artery: A Cadaveric Anatomical Study

**DOI:** 10.7759/cureus.9897

**Published:** 2020-08-20

**Authors:** Eleni Panagouli, Ioannis Antonopoulos, Gregory Tsoucalas, Alexandros Samolis, Dionysios Venieratos, Theodore Troupis

**Affiliations:** 1 Department of Anatomy, Medical School, National and Kapodistrian University of Athens, Athens, GRC; 2 Department of Anatomy, Medical School, Democritus University of Thrace, Alexandroupolis, GRC

**Keywords:** aortic arch, common carotid artery, torso length, height, innominate artery

## Abstract

Introduction

The brachiocephalic artery (BCA) is the largest branch that arises from the aortic arch, which varies in length. The present study focuses on BCA length and its probable correlation with height and torso length.

Methods

The BCA length (from the artery’s origin to the arcus aortae), the length of the torso, and height were measured in 76 embalmed adult human cadavers of Caucasian (Hellenic) origin.

Results

A total of 74 arteries were measured (36 females and 38 males). The mean length was found to be 3.82 cm (SD=±0.947, SE=0.110). In male cadavers, the mean BCA length was 3.94 cm (SD=±0.980, SE=0.159) and in females, it was found to be 3.69 cm (SD=±0.905, SE=0.151). No statistically significant difference was found (p=0.248, p>0.05) The mean torso length was 62.27 cm (SD=±4.325, SE=0.496) and the mean height was 155.3 cm (SD=±10.124, SE=1.161). The BCA length was correlated with body height and torso length in both sexes. A statistically significant correlation was found only between BCA length and body height in male cadavers (r=0.267, p=0.021).

Conclusion

The morphometric characteristics of the BCA are of great importance in a number of surgical procedures, such as stenting and catheterization in cases of aneurysms. One statistically significant correlation was observed in our study, which could be considered an important finding, as it could lead to the plausible assumption that greater height leads to the formation of larger arteries.

## Introduction

The brachiocephalic artery (BCA), also known as the brachiocephalic trunk or innominate artery, is the largest branch that arises from the aortic arch. It begins its course at the level of the second right costal cartilage before the insertion of the left common carotid artery (CCA) [1]. Afterward, it follows an oblique, ascending trajectory until it reaches the upper border of the right sternoclavicular articulation and then divides into the right subclavian and right CCA. Except from these two arteries, the BCA usually gives off no other sided branch. Rarely, a thyroidea ima or a small thymic or bronchial branch may arise from the BCA. In case the aorta arches over to the right side, the BCA follows a course toward the left side of the neck instead of the right [1].

Referring to the usual position and topographic relations of the BCA, the artery runs behind the sternal manubrium from which it is separated by the origins of the sternohyoid and sternothyroid muscles, the remains of the thymus, and, finally, the right inferior thyroid and left brachiocephalic veins. The left CCA ascends on its left side, descends the left inferior thyroid vein, and on its right side, descends the right innominate vein as well as the right vagus nerve. Ultimately, the BCA runs in front of the anterior wall of the trachea [1-2].

Several variations have been reported regarding the point from which the BCA arises, the pulmonary trunk being an extremely rare case [3]. The existence of two BCAs is also reported [4] and even the total absence of it. In the latter case, the right common carotid and subclavian arteries arise directly from the aortic arch.

The BCA also varies in length. This is normally expected and occurs obviously in other arteries as well, which also vary in length before their final bifurcation. In a previous study, the lengths of several arteries had been measured and correlated in a number of human cadavers [5]. The present paper constitutes a continuation but with a greater sample and a special focus on BCAs length and its correlation with height and torso length.

## Materials and methods

Seventy-six embalmed adult human cadavers of Caucasian (Hellenic) origin (39 males and 37 females) were subjected to routine educational dissection at the anatomy department of the Medical School of the University of Athens. The cadavers were derived from body donation with informed consent, written and signed (with signature authentication) by the donors themselves [6]. The protocol for the present research had been approved by the ethics committee of our institution. The age of the specimens ranged between 39 and 98 years (average age 74.9 years, SD=± 11.230, SE=1.288).

In each cadaver that underwent dissection, the BCA was dissected and measured. The length of the BCA was evaluated from its origin to its bifurcation to the right CCA and the right subclavian artery. In addition, the torso length and the height of each cadaver were measured. As “torso length,” the distance between the hyoid bone and the pubic symphysis was defined. The distance between the bregma and the heel was measured as "height." All the distances were measured by calipers. In order to obtain the distances between the branches of the vessels, the center of each vessel was taken as the recordable point of its origin.

Statistical analysis

The results were recorded in the form of tables and then were subjected to statistical analysis with the purpose of calculating the average, maximum, and minimum value, standard deviation (SD), standard error of the mean (SE), and, finally, the correlations between the discovered distances. In order to correlate the measured arterial distances and lengths, Pearson’s correlation coefficient (r) was used. For comparing continuous variables, the t-test was applied and for nominal variables, the x2 test. Statistical analysis was done using Statistical Package for the Social Sciences (SPSS) 15.0 (SPSS Inc, Chicago, IL).

## Results

The BCA was identified and dissected in all cadavers mentioned above except in two; in one cadaver (female), both the BCA and subclavian artery were destroyed during embalming and in the other one (male), the BCA was absent. As a result, a total of 74 arteries were measured (36 females and 38 males).

The BCA length was measured from the artery’s origin to the arcus aortae till its division into the right subclavian artery (SCA) and right common carotid artery. The mean length was found to be 3.82 cm (SD=±0.947, SE=0.110), with a minimum length of 1.7 cm and a maximum of 6 cm (Table 1). In male cadavers, the mean BCA length was 3.94 cm (SD=±0.980, SE=0.159) and, therefore, it was slightly larger than the corresponding in females, which was found to be 3.69 cm (SD=±0.905, SE=0.151) (Table 1). Although the measurements differed between the two genders, no statistically significant difference was found (p=0.248, p>0.05).

**Table 1:** Presentation of measurements *BCA: Brachiocephalic artery

	Mean (cm)	Min (cm)	Max (cm)	SD (cm)	SE
BCA length (n=74)	3.82	1.7	6.0	±0.947	0.110
male (n=38)	3.94	2.0	6.0	±0.980	0.159
female (n=36)	3.69	1.7	6.0	±0.905	0.151
Torso height (n=76 cadavers)	62.27	51.5	70.7	±4.325	0.496
male (n=39)	64.78	59.5	70.7	±3.249	0.520
female (n=37)	59.62	51.5	67	±3.709	0.610
Body height(n=76 cadavers)	155.3	128	174.5	±10.124	1.161
male (n=39)	162.1	146	174.5	±7.043	1.279
female (n=37)	148.1	128	162	±7.571	1.245

**Figure 1:**
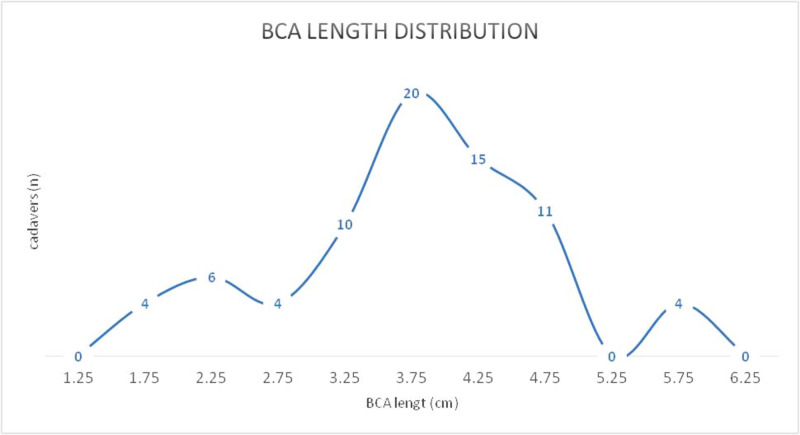
Distribution of BCA length in a total of 76 dissected cadavers BCA: brachiocephalic artery

The total specimens' BCA length distribution is shown in Figure 1.

The length of the torso, i.e., the distance between the hyoid bone and the pubic symphysis was also measured in every cadaveric specimen (76 in total; 39 males and 37 females).

**Figure 2:**
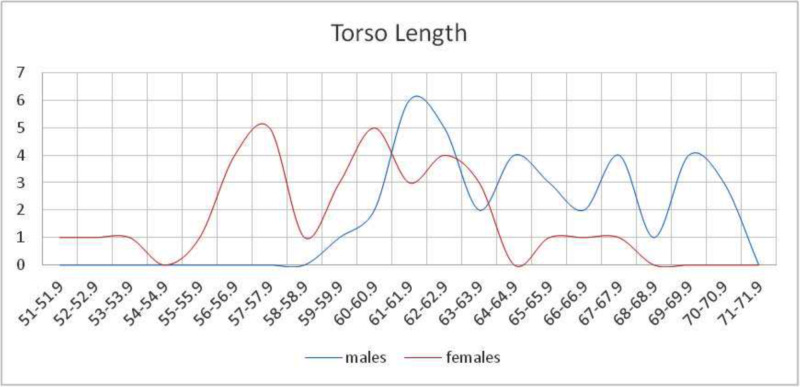
Distribution of the torso length (cm) in both sexes A statistically significant difference was observed (p<0.001).

The mean torso length was 62.27 cm (SD=±4.325, SE=0.496), with a minimum of 51.5 cm and a maximum of 70.0 cm (Table 1). A statistically significant difference was found between the two sexes (p<0.001). The torso length distribution (cm) is shown in Figure 2.

**Figure 3:**
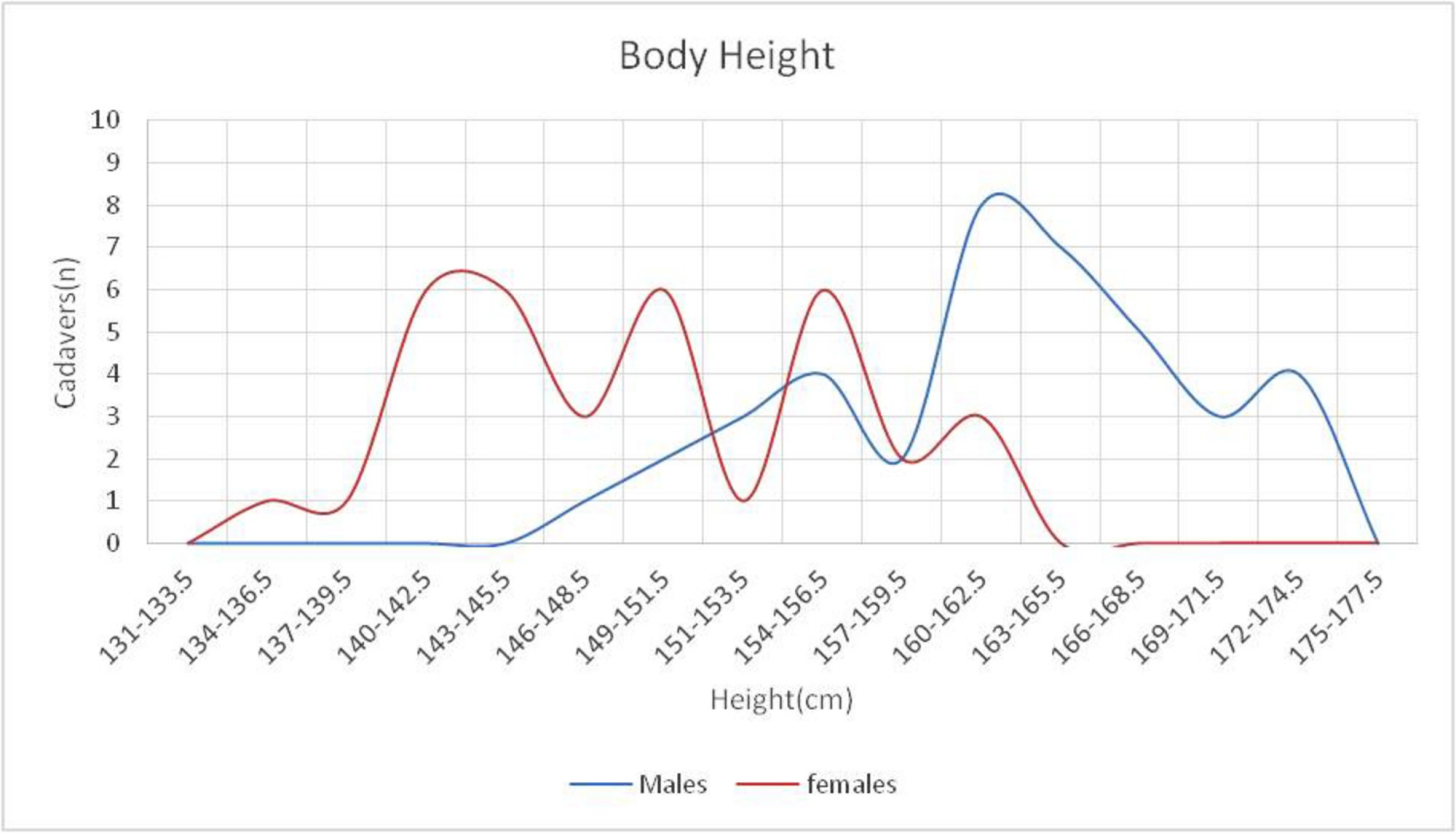
Distribution of body height in both sexes A statistically significant difference was observed (p<0.001).

The body height, i.e., the distance between the bregma and the calcaneus, was also measured in all dissected cadavers. The mean height value was 155.3 cm (SD=±10.124, SE=1.161), with a minimum of 128.0 cm and a maximum of 174.5 (Table 1). The body height between the two sexes is depicted in Figure 3. A statistically significant difference was found between the two sexes (p<0.001).

BCA length was correlated with body height and torso length in both sexes. A statistically significant correlation was found only between BCA length and body height and only in male cadavers (r=0.267, p=0.021).

## Discussion

The aortic arch constitutes the continuation of the ascending aorta and normally begins with the origin of the BCA [7]. According to classical anatomical references, the BCA is the first of the three main branches of the aortic arch with a length varying between 4 cm and 5 cm and a diameter of 12.1 mm ± 1.6 mm [1-2,8]. The morphometric characteristics of the BCA might be rather useful in a number of surgical procedures such as stenting and catheterization in cases of aneurysms [8]. To our knowledge, there are numerous studies dealing with the BCA’s diameter [7,9-14] but only a few comparative data exist concerning its length [15-16].

Honig et al. (1952) mention in their study that the expected length of the BCA ranges from 3.7 to 5.0 cm and a concomitant lengthening occurs when arteriosclerosis coexists [15]. The authors cite that the aforementioned length was observed in postmortem studies with no further details. Shin et al. (2008) [16] performed a study on 25 Korean adult cadavers (17 males and eight females, mean age of 63 years) and among other data, they measured the distance from the origin of the BCA to the origin of the right CCA, which corresponds to the length of BCA. The mean distance was 3.25 mm with a range between 2.42 and 4.6 cm [15]. A greater mean length was observed in the present study (mean length 3.82±0.947 cm, SE=0.110) as well as a greater range, with the minimum length being 1.7 cm and the maximum being 6 cm. 

Concerning correlations between lengths, only one statistically significant correlation was observed between BCA length and body height in male cadavers only (r=0.267, p=0.021). That probably occurred because males presented a greater range of values in both BCA length and height (Table 1). Nevertheless, we consider this an important finding, as it could lead to the plausible assumption that greater height leads to larger arteries.

The typical branching pattern of the aortic arch with the BCA, left subclavian, and left CCA has been classified as the normal pattern type A (Adachi’s classification) [17]. This branching pattern occurs only in 70% of the cases [18]. A series of anatomical variations have been reported, such as an abnormal branching pattern of the aortic arch itself or the BCA, a left BCA, a right aortic arch, vascular agenesis, etc. [18]. In the present series, only a case of absence of the BCA was observed (1/76 - 1.3%). The corresponding incidence according to the available literature is 0.44% [8]. No further variations were discovered.

Thorough knowledge of the aortic arch and its branches is important for surgeons, pathologists, and invasive radiologists. Serious conditions, such as aortic aneurysms, aortic dissection, coronary artery ectasia, or stenosis, might occur at this site and all of them need immediate diagnosis and treatment [7,11,16]. Lately, surgical techniques in these cases involve the use of a catheter, which is entered through the femoral artery and is moved upward through the abdominal aorta to the aortic arch. The length of this catheter is evaluated according to the torso length. Knowledge of the length range of the BCA might be helpful, along with the fact that a statistically significant correlation was observed between BCA length and the body height in males. Additionally, an assortment of the BCA length might facilitate radiologists, as computed tomography (CT) and magnetic resonance imaging (MRI) angiograms are commonly used for the diagnosis of conditions of the aortic arch and for guidance during surgical procedures.

## Conclusions

The morphometric characteristics of the BCA, such as its length, are of great importance in a number of surgical procedures such as stenting and catheterization in cases of aneurysms. Concerning correlations between lengths, only one statistically significant correlation was observed in our study, which could be considered an important finding, as it could lead to the plausible assumption that greater height leads to larger arteries.
